# Analysis of cardiac arrest after coronary artery bypass grafting

**DOI:** 10.1186/s13019-024-02963-w

**Published:** 2024-07-16

**Authors:** Tengjiao Yang, Xieraili Tiemuerniyazi, Zhan Hu, Wei Feng, Fei Xu

**Affiliations:** Department of Cardiovascular Surgery, National Center for Cardiovascular Disease and Fuwai Hospital, Chinese Academy of Medical Sciences, Peking Union Medical College, 167 Beilishi Road, Xicheng, Beijing, 100037 People’s Republic of China

**Keywords:** Cardiac arrest, Resuscitation, Shockable rhythm, General ward, Prognosis

## Abstract

**Background:**

Cardiac arrest after coronary artery bypass grafting (CABG) is a serious complication with low survival rate. The prognosis of patients with cardiac arrest in the general ward is worse than that in the intensive care unit (ICU) because of the delayed and poor rescue conditions.

**Methods:**

This retrospective study included patients who experienced cardiac arrest after CABG surgery between January 2010 and December 2019 at the Fuwai Hospital. Differences in cardiac arrest between the ICU and the general ward were compared. The patients were divided into shockable and non-shockable rhythm groups, and the differences between the two groups were compared. Finally, we proposed a management protocol for cardiac arrest in the general ward.

**Results:**

We retrospectively analyzed 41,450 patients who underwent CABG only, of whom 231 (0.56%) experienced cardiac arrest post-surgery in the ICU (185/231) or in the general ward (46/231). The rescue success rate and 30-day survival rate of the patients with cardiac arrest in the general ward were 76.1% (35/46) and 58.7% (27/46), respectively. The incidence of the different arrhythmia types of cardiac arrest in the general ward compared with that in the ICU was different (*P* = 0.010). The 30-day survival rate of the non-shockable rhythm group was 31.8% (7/22), which was worse than that of the shockable rhythm group (83.3% [20/24]; *P* = 0.001). Kaplan–Meier survival analysis showed that the prognosis of the non-shockable group was poor (*P* < 0.001).

**Conclusions:**

The incidence of cardiac arrest after CABG was low. The prognosis of patients in the general ward was worse than that of those in the ICU. The proportion of non-shockable rhythm type cardiac arrest was higher in the general ward than in the ICU, and patients in this group had a worse early prognosis.

## Introduction

Coronary artery bypass grafting (CABG) is the main revascularization strategy for patients with multi-vessel or complex coronary artery disease with comorbidities, such as diabetes, renal dysfunction, and left ventricular systolic dysfunction [1]. The mortality rate of elective CABG surgery ranges from 1 to 3% [2]. Cardiac arrest is a serious postoperative complication of CABG and is associated with a significantly low in-hospital survival rate of 30–79% [3–5]. Older age, low ejection fraction, and preoperative myocardial infarction are risk factors for poor prognosis [6]. The common causes of post-cardiac surgery arrest are graft failure, tamponade, and hemorrhage, and most causes are correctable or reversible [5, 7–9]. Cardiac arrest usually occurs during the early postoperative period in the intensive care unit (ICU), and patients can generally be rescued in time. However, a small number of cardiac arrests occur after patients are transferred out of the ICU [7, 10]. Owing to delayed rescue, poor rescue conditions, and lack of medical personnel in the general ward, the prognosis of patients who experience cardiac arrest in the general ward is worse than that in the ICU. In this study, we reviewed and analyzed cases of cardiac arrest after CABG in our hospital. We compared the differences in cardiac arrest between the general ward and ICU and proposed a management protocol in the general ward.

## Patients and methods

### Ethical statement

The Ethics Committee of Fuwai Hospital approved the use of the patients’ clinical data on March 3rd, 2022 (Approval No.: 2021 − 1644) and waived the requirement to obtain individual informed consent for the publication of this study. All procedures and management implemented in this study, involving human participants, were in accordance with the ethical standards of the Institutional and National Research Committee and the 1975 Helsinki Declaration and its later amendments or comparable ethical standards.

### Patients

We retrospectively studied patients who underwent CABG between January 2010 and December 2019 at the Fuwai Hospital. Inclusion and exclusion criteria: Patients who underwent CABG surgery only, those with cardiac arrest occurring in the ICU or general ward post-surgery, and those with cardiac arrest occurring in other places were excluded.

Surgery was performed using either the cardiopulmonary bypass (CPB) or the off-pump method. Temporary pacemaker leads were routinely placed in the epiventricular region. After extubation, patients were transferred to the general ward if circulation was stable and there were no major complications. Continuous ECG monitors were used for all patients in the ICU and general ward.

On the other hand, there are four different rhythm types of cardiac arrest: ventricular fibrillation and pulseless ventricular tachycardia, pulseless electrical activity, cardiac asystole, and extreme bradycardia [11]. Ventricular fibrillation and pulseless ventricular tachycardia are shockable rhythms, while others are non-shockable rhythms. Different resuscitation processes for shockable and non-shockable cardiac arrest have been recommended in the literature [12, 13]. Therefore, we divided the patients into shockable and non-shockable groups and compared the differences between the two groups in the ICU and general ward.

**Fig. 1:**
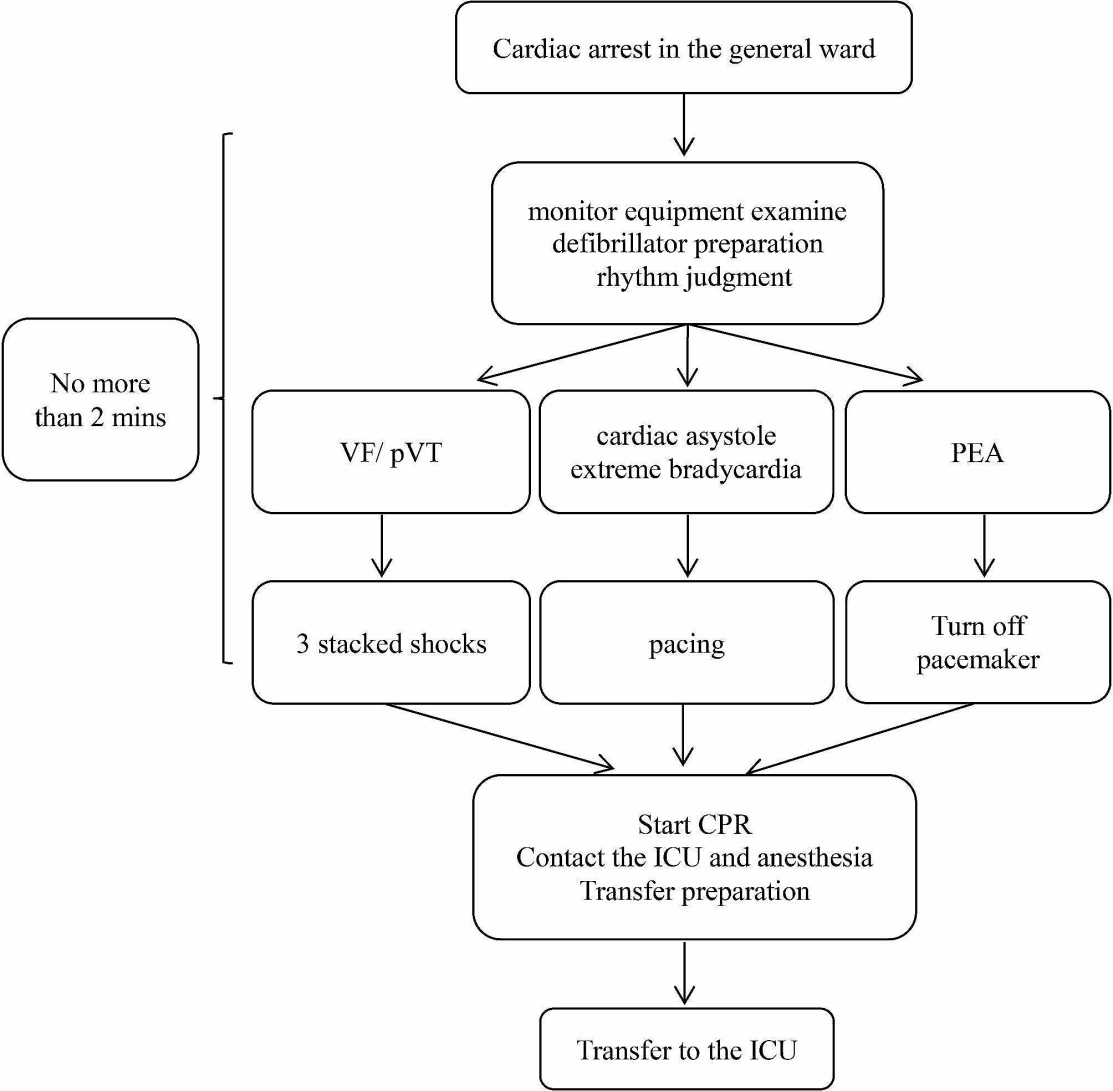
Management protocol for cardiac arrest in the general ward. CPR, cardiopulmonary resuscitation; ICU, intensive care unit; PEA, pulseless electrical activity; VF, ventricular fibrillation; pVT, pulseless ventricular tachycardia

In the ICU, we managed the arrest mainly based on the protocol of The Society of Thoracic Surgeons expert consensus [11]. In the general ward, we proposed our management protocol, as shown in Fig. 1. Multiple medical staff members are required to work cooperatively in teams. When hearing the cry for help, doctors and nurses compose a rescue team. During the first minute, a series of tasks should be conducted, such as examination of the monitoring equipment, defibrillator preparation, rhythm judgment, and defibrillation, if necessary. Subsequent work involved starting CPR, contacting the ICU and anesthetist, and preparing for transfer to the ICU. Successful rescue was defined as the recovery of stable autonomous circulation or stable circulation with IABP or ECMO assistance, and CPB was removed.

### Data collection

We investigated each patient’s medical data (including progress notes, nursing records, medical advice, and other reports), and recorded their general clinical data, preoperative cardiac function, surgery-related information, time of cardiac arrest after surgery, cardiac arrest rhythm type, rescue process, and outcomes. Data on patient survival status and cardiac function were obtained through telephone connections with the patients’ families and outpatient follow-ups. The follow-up ended if the patient died or until February 1, 2023, the latest follow-up.

### Statistics and analysis

IBM SPSS Statistics 25 (IBM Corp., Armonk, NY, USA) was used for statistical analysis. Data with a normal distribution are presented as mean ± standard deviation. The t-test was used to compare the means between groups of normally distributed data, and the Mann–Whitney U test was used to compare groups of non-normally distributed data. Numerical data were presented as the number and percentage of cases, and comparisons between groups were performed using the chi-square test. Survival rates were compared using the Kaplan–Meier method. All statistical tests were two-sided, and *P* < 0.05 was considered statistically significant.

First, we compared the baseline and post-arrest data of the patients in the ICU and general wards. Subsequently, differences between the shockable and non-shockable groups were compared. To determine the prognostic difference between the two groups, we analyzed the causes of cardiac arrest and used the Kaplan-Meier method to compare the midterm and long-term outcomes.

## Results

A total of 41,450 patients underwent isolated CABG at our hospital between January 2010 and December 2019. Among these patients, 231 (0.56%) developed cardiac arrest postoperatively in the ICU or general ward and 46 (0.11%) of these arrests occurred in the general ward. Table 1 shows a comparison of the baseline data of patients with cardiac arrest in the general ward and the ICU. The types of cardiac arrest were similar between the groups; however, the proportions of the different types of cardiac arrest differed significantly (*P* = 0.006). In our study, 47.9% (22/46) of patients in the general ward had a non-shockable rhythm, which was significantly higher than that in the ICU. Patients with cardiac arrest in the general ward had worse preoperative cardiac function (*P* = 0.005) and a higher proportion of previous myocardial infarction (*P* = 0.027) than those with cardiac arrest in the ICU. The rescue and 30-day survival rates of patients in the general ward were lower than those of patients in the ICU; however, the difference was not statistically significant (*P* = 0.181 and *P* = 0.153, respectively).

**Fig. 2:**
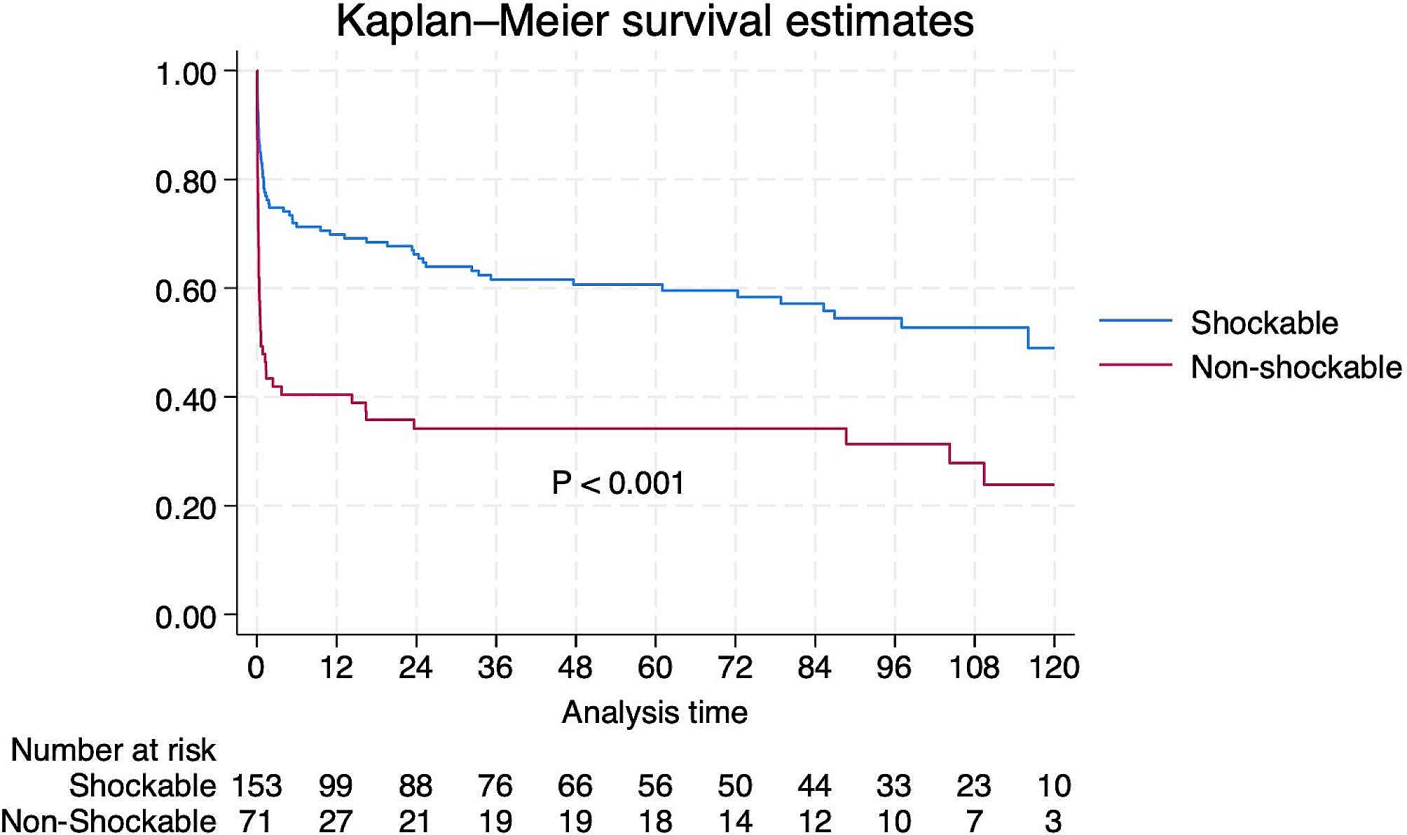
Survival curve by types of cardiac arrest (shockable group and non-shockable group)

Patients were divided into shockable and non-shockable groups according to the type of arrhythmia during cardiac arrest. Baseline patient data are shown in Table 2. In the general ward, the preoperative left ventricular end-diastolic diameter (LVEDd) was larger and the preoperative left ventricular ejection fraction (LVEF) was lower in the shockable group than in the non-shockable group (*P* = 0.005 and *P* = 0.001, respectively). We also found the above tendency in the ICU, although the differences were not significant (*P* = 0.105 and *P* = 0.134). The prognosis was poor in the non-shockable group, the rescue success rate was lower (*P* = 0.09 in the general ward; *P* = 0,005 in the ICU) and the 30-day survival rate after surgery was significantly lower (*P* = 0.001 and *P* = 0.000, respectively). As shown in Fig. 2, the early survival rate was significantly worse in the non-shockable group; however, the mid- and long-term survival rates were stabilized. The causes of cardiac arrest in the two groups are shown in Table 3, and they differed significantly between the groups (*P* = 0.001). The clear causes in the shockable group were mostly myocardial ischemia or poor cardiac function, whereas the rates of other causes, such as bleeding or electrolyte disturbance, increased in the non-shockable group.

## Discussion

Cardiac arrest after cardiac surgery is uncommon, but can be life-threatening, with a reported incidence of 0.7–2.9% [14]. In this study, the incidence of cardiac arrest after CABG in our center was 0.56%, which is lower than that in previous studies [15]. The incidence of cardiac arrest in the general ward (0.11%) was much lower than the overall rate. However, the prognosis for patients with cardiac arrest after CABG in the general ward is poor. A previous study showed that the rescue success rate for patients who experienced cardiac arrest in the ICU early after cardiac surgery was 80% [14]. Our hospital data showed that the rescue success rate in the ICU was 84% and that in the general ward was 76%. However, the survival rate of patients in the general ward after cardiac arrest (only 58.7% at discharge) was much worse than that of patients in the ICU, and the lower survival rate was similar to that reported in literature [5].

The factors associated with the poor prognosis of patients in the general ward after cardiac arrest varied. This study showed that patients in the general ward with cardiac arrest had worse preoperative cardiac function and a higher proportion had previous myocardial infarction. These patients also tend to develop various arrhythmias that induce cardiac arrest in the early postoperative period. Patients with poor cardiac function are sensitive to circulation volume, electrolyte disturbances, or infections. Additionally, nurse and patient ratios differed greatly between the general ward and the ICU. The frequency of laboratory monitoring and the intensity of vital sign monitoring in the general wards were relatively low. These unfavorable factors may lead to the late detection of cardiac arrest and limit rescue measures. The literature suggests that insufficient personnel is a factor for poor prognosis [8]. Thus, a mature rescue protocol can improve the rescue success rate and prognosis. In this study, there was no statistically significant difference in the survival rate between the general ward and the ICU, suggesting that the rescue protocol we proposed was effective.

Different types of cardiac arrests in general wards were associated with different early and long-term outcomes [16]. Compared with patients in the ICU, the proportion of non-shockable cardiac arrests was higher, and the rescue and 30-day survival rates were significantly lower. We inferred that the causes, such as bleeding or electrolyte disturbance, were not easy to detect or correct quickly in the general ward and were apt to induce non-shockable arrest. However, when encountered with these factors, the ECG has a dynamic progress, which provides an opportunity to treat the reversible causes of pre-arrest [17–19]. Serious bradycardia results in cardiac congestion and an excessive blood volume. These factors exacerbate the condition and lead to poor rescue outcomes. Survival curves showed that the non-shockable group had a higher early mortality rate than that of the shockable group. However, the long-term survival rate of the non-shockable group tended to be stable. Our results suggest that patients in the non-shockable group had relatively good preoperative cardiac function; therefore, discharged patients experienced fewer cardiovascular events during the follow-up. Considering the poor early outcomes in the non-shockable group, the most important measure to improve prognosis is to avoid cardiac arrest by prophylactically addressing correctable or reversible causes [8, 18, 20]. Additionally, the use of safety checklists and rapid response teams can help prevent cardiac arrest after cardiac surgery [8, 9, 21].

When cardiac arrest occurs in the general ward, it is not always detected quickly, and surgical measures are difficult to implement. No effective resuscitation guidelines are currently available for this situation; therefore, the ideal first treatment measure for cardiac arrest remains unclear. For patients who develop cardiac arrest in the ICU after cardiac surgery, immediate chest compression is not recommended in the literature [9, 22, 23]. Instead, the type of arrhythmia should be determined, reversible causes should be corrected, and electrical defibrillation or pacing should be performed immediately [9, 24]. Notably, many causes of sudden arrest after cardiac surgery are reversible or correctable, and most patients can be saved after timely treatment. Additionally, the wards were equipped with defibrillation and temporary pacing equipment, which can be quickly obtained to perform defibrillation or pacing therapy. Moreover, many patients with shockable rhythm can convert to normal rhythm after defibrillation, thus avoiding secondary injury caused by external chest compressions. Previous studies have shown that the incidence of complications associated with external chest compressions during cardiopulmonary resuscitation is very high, especially after cardiac surgery, potentially leading to fatal injuries such as rupture of the papillary muscle and left ventricle [25]. In our study, one patient (2.7%) developed rupture of the left ventricular free wall as a result of chest compressions. Chest compression has little effect on the rescue success rate and short-term prognosis after surgery. However, performing chest compressions delays the time for defibrillation conversion or pacing treatment, and increases the risk of chest compression-associated injuries. Therefore, we recommend electric defibrillation or temporary cardiac pacing immediately after sudden cardiac arrest is detected in the general ward; if this is unsuccessful, cardiopulmonary resuscitation should be started immediately.

Considering the risks associated with external cardiac compressions and the reversibility of the cause of cardiac arrest after cardiac surgery, reopening after cardiac arrest in the ICU is recommended as soon as possible after defibrillation or pacing failure [11, 14]. However, the survival rate after chest reopening in the general ward is almost zero [26]. Previous studies have recommended against chest reopening in the general ward in patients with cardiac arrest. The only possible effective measure is to immediately transfer these patients to the ICU for reopening after detecting cardiac arrest [7, 26]. In this study, 10 (21.7%) patients in the general ward group underwent chest reopening, and one patient in the general ward did not survive. The other nine patients underwent chest reopening in the ICU or operating room; of these patients, three survived and were discharged, and one developed neurological complications. Therefore, the key to the successful rescue of patients with indications for chest reopening is to quickly transfer the patient to the ICU or operating room. Emergency transfer should be performed during chest compressions, and the ICU or the operating room should be informed to prepare for reopening [22]. All the works are urgent, a well-trained rapid response team can improve the rescue success rate and decrease the mortality rate [8].

## Limitations

This study had several limitations. The main limitation was the retrospective design, which created difficulties in acquiring specific clinical details of the events preceding each cardiac arrest and the acute characteristics of the arrest. The second limitation was the small number of patients, which underpowered the study for subgroup comparisons. This is also why we did not present more details of peri-arrest management. Additional limitations include single-center and single-disease settings, which reduce the generalizability of our results to broader clinical practice.

**Table 1:** Baseline data of the patients in the general wards and ICU

Variable	General ward (*n* = 46)	ICU (*n* = 185)	*P*
Age (years), mean ± SD	64.9 ± 8.5	62.9 ± 8.9	0.18
Female, no (%)	15 (32.6%)	32 (17.3%)	0.021
Hypertension, no (%)	32 (69.6%)	129 (69.7%)	0.983
Renal dysfunction, no (%)	6 (13%)	19 (10.3%)	0.588
Diabetes mellitus, no (%)	21 (45.7%)	60 (32.4%)	0.172
Peripheral vascular disease, no (%)	10 (21.7%)	46 (24.9%)	0.658
NYHA III or IV, no (%)	18 (39.1%)	36 (19.5%)	0.005
CCS III or IV, no (%)	21 (45.7%)	66 (35.7%)	0.211
Previous myocardial infarction, no (%)	28 (60.9%)	79 (42.7%)	0.027
Previous acute myocardial infarction, no (%)	11 (23.9%)	21 (11.4%)	0.027
LVEDD (mm), mean ± SD	51.4 ± 6.8	51.3 ± 7.3	0.92
LVEF (%), mean ± SD	53.7 ± 13	57.1 ± 12	0.087
EUROSCORE II, mean ± SD	2.94 ± 4.33	2.55 ± 3.39	0.52
Number of grafts, mean ± SD	3.26 ± 1.02	2.97 ± 0.97	0.076
On-pump operation, no (%)	26 (56.5%)	102 (55.1%)	0.886
CPB time (min), mean ± SD	117.19 ± 42.06	115.32 ± 63	0.886
Aortic cross-clamp time (min), mean ± SD	78.44 ± 31.56	71.78 ± 28.7	0.314
Operation time (min), mean ± SD	234.09 ± 62.38	238.55 ± 114	0.799
Postoperative hospital stay (d), mean ± SD	20.11 ± 25.72	14.81 ± 23	0.174
Duration between the end of surgery and onset of cardiac arrest (hour), mean ± SD	113.15 ± 68.1	50.84 ± 140.3	0.004
Types of cardiac arrest			
Ventricular fibrillation or tachycardia, no (%)	24 (52.2%)	133 (71.9%)	0.006
Pulseless electrical activity, no (%)	1 (2.2%)	11 (5.9%)	
Bradycardia or asystole, no (%)	21 (45.7%)	41 (22.2%)	
Chest reopening, no(%)	10 (21.7%)	74 (40%)	0.021
Successful resuscitation, no (%)	35 (76.1%)	157 (84.9%)	0.181
Time to successful resuscitation (min), mean ± SD	85.5 ± 157.8	149 ± 247.3	0.059
Survival rate 30 days after surgery, no (%)	27 (58.7%)	129 (69.7%)	0.153
Survival after chest reopening, no(%)	2(20%)	39(52.7%)	0.089

Data are presented as mean ± standard deviation or n (%). CCS, Canadian Cardiovascular Society; CPB, cardiopulmonary bypass; ICU, intensive care unit; LVEDD, left ventricular end-diastolic diameter; LVEF, left ventricular ejection fraction; NYHA, New York Heart Association

**Table 2:** Comparison of the different groups in the general wards and ICU by the type of cardiac arrest

Variable	General Wards		*P*	ICU		*P*
	Shockable group *n* = 24	Non-Shockable group *n* = 22		Shockable group *n* = 133	Non-Shockable group *n* = 52	
Age (years), mean ± SD	62.9 ± 8.7	66.9 ± 8.2	0.117	62.7 ± 8.8	63.6 ± 9.3	0.536
Female, no (%)	9 (37.5%)	6 (27.3%)	0.460	20 (15%)	12 (23.1%)	0.194
Hypertension, no (%)	14 (58.3%)	18 (81.8%)	0.084	90 (67.7%)	39 (75%)	0.329
Renal dysfunction, no (%)	4 (16.7%)	2 (9.1%)	0.667	14 (10.5%)	5 (9.6%)	0.854
Diabetes mellitus, no (%)	10 (41.7%)	11 (50%)	0.571	42 (31.6%)	18 (34.6%)	0.692
Peripheral vascular disease, no (%)	4 (17.4%)	6 (27.3%)	0.491	33 (24.8%)	13 (25%)	0.979
NYHA III or IV, no (%)	9 (37.5%)	9 (40.9%)	0.813	23 (17.3%)	13(25%)	0.234
CCS III or IV, no (%)	11 (45.8%)	10 (45.5%)	0.979	48(36.1%)	18(34.6%)	0.851
Previous myocardial infarction, no (%)	17 (70.8%)	11 (50%)	0.148	61 (45.9%)	18 (34.6%)	0.164
Previous acute myocardial infarction, no (%)	6 (25%)	5 (22.7%)	0.857	15 (11.3%)	6 (11.5%)	0.960
LVEDD (mm), mean ± SD	54.3 ± 7.2	48.62 ± 5.2	0.005	51.9 ± 7.7	49.9 ± 6.0	0.105
LVEF (mm), mean ± SD	47.6 ± 13.2	60.3 ± 9.5	0.001	56.3 ± 13.5	59.2 ± 9.1	0.134
EUROSCORE II, mean ± SD	3.60 ± 5.44	2.27 ± 2.60	0.288	2.58 ± 3.38	2.49 ± 3.47	0.867
Average number of grafts, mean ± SD	3.3 ± 1.1	3.2 ± 0.9	0.841	3.0 ± 1.0	2.9 ± 0.9	0.347
On-pump operation, no (%)	13 (54.2%)	13 (59.1%)	0.736	73 (54.9%)	29 (55.8%)	0.914
CPB time (min), mean ± SD	107.6 ± 33.6	126.8 ± 48.5	0.254	113.2 ± 47.4	120.7 ± 92.2	0.590
Aortic cross-clamp time (min), mean ± SD	80.2 ± 30.6	78.7 ± 33.3	0.903	72.0 ± 30.4	75.8 ± 40.2	0.608
Operation time (min), mean ± SD	225.1 ± 56.6	243.9 ± 68.1	0.312	235.4 ± 87.7	246.6 ± 164.6	0.552
Postoperative hospital stay (d), mean ± SD	25.5 ± 33.8	14.2 ± 9.6	0.136	13.7 ± 10.4	17.6 ± 40.3	0.309
Duration between the end of surgery and onset of cardiac arrest (hour), mean ± SD	111.8 ± 71.7	114.6 ± 65.6	0.889	48.4 ± 135.7	57.1 ± 152.7	0.707
Time to successful resuscitation (min), mean ± SD	75.5 ± 158.2	100.4 ± 161.8	0.654	111.4 ± 228.6	266.6 ± 269.1	0.001
Successful resuscitation, no (%)	21 (87.5%)	14 (66.7%)	0.09	119 (89.5%)	38(73.1%)	0.005
Survival rate 30 days after surgery, no (%)	20 (83.3%)	7 (31.8%)	0.001	104 (78.2%)	26 (50%)	0.000
Survival without Neurological complication, no (%)	15 (62.5%)	6 (27.3%)	0.017	100 (75.2%)	20 (38.5%)	0.000

Data are presented as mean ± standard deviation, n (%). CCS, Canadian Cardiovascular Society; CPB, cardiopulmonary bypass; d, days; LVEDD, left ventricular end-diastolic diameter; LVEF, left ventricular ejection fraction; no, number; NYHA, New York Heart Association; SD, standard deviation

**Table 3:** Causes of cardiac arrest

Cause	Shockable Group (*n* = 157)	Non-Shockable Group (*n* = 74)	*P*
Myocardial ischemia	49(31.2%)	18 (24.3%)	0.001
Bleeding/ Cardiac temponade	5 (3.2%)	15 (20.3%)	
Electrolyte disturbance	11 (7.0%)	6 (8.1%)	
Respiratory dysfunction	5 (3.2%)	7 (9.5%)	
Cardiac dysfunction	46 (29.3%)	15 (20.3%)	
Infection	6 (3.8%)	5 (6.8%)	
Unknown	35 (22.3%)	8 (10.8%)	

Data are presented as n (%)

## Conclusions

In the present study, the incidence of cardiac arrest after CABG was low. The prognosis of patients in the general ward was worse than that of patients in the ICU. The proportion of non-shockable rhythm type of cardiac arrest was higher in the general ward than in the ICU, and the patients in this group had a worse early prognosis.

## Data Availability

No datasets were generated or analysed during the current study.
